# Phenotypic variability within the desminopathies: A case series of three patients

**DOI:** 10.3389/fneur.2022.1110934

**Published:** 2023-01-16

**Authors:** Dennis Yeow, Matthew Katz, Robert Henderson, Sandhir Prasad, Russell Denman, Stefan Blum, Mark Davis, Thomas Robertson, Pamela McCombe

**Affiliations:** ^1^Department of Neurology, Royal North Shore Hospital, Sydney, NSW, Australia; ^2^Department of Neurology, Royal Brisbane and Women's Hospital, Brisbane, QLD, Australia; ^3^Department of Cardiology, Royal Brisbane and Women's Hospital, Brisbane, QLD, Australia; ^4^Department of Cardiology, The Prince Charles Hospital, Brisbane, QLD, Australia; ^5^Department of Neurology, Princess Alexandra Hospital, Brisbane, QLD, Australia; ^6^Department of Diagnostic Genomics, Pathwest Laboratory Medicine, Perth, WA, Australia; ^7^Department of Pathology, Royal Brisbane and Women's Hospital, Brisbane, QLD, Australia

**Keywords:** desmin, desminopathy, cardiomyopathy, myofibrillar myopathy, splice-site mutation

## Abstract

The *DES* gene encodes desmin, a key intermediate filament of skeletal, cardiac and smooth muscle. Pathogenic *DES* variants produce a range of skeletal and cardiac muscle disorders collectively known as the desminopathies. We report three desminopathy cases which highlight the phenotypic heterogeneity of this disorder and discuss various factors that may contribute to the clinical differences seen between patients with different desmin variants and also between family members with the same variant.

## 1. Introduction

Desmin is an intermediate filament which forms links between myofibrils, desmosomes, the nuclear envelope and the sarcolemma within skeletal, cardiac and smooth muscle (1). Structurally it consists of a head domain, a central rod domain with 1A, 1B, 2A, and 2B subunits and a tail domain. Mutations in the desmin gene (*DES*) produce a wide range of phenotypes collectively termed the desminopathies which can present with any combination of skeletal myopathy (myofibrillar myopathy, limb-girdle muscular dystrophy, Kaeser's scapuloperoneal syndrome), cardiomyopathy (dilated cardiomyopathy, hypertrophic cardiomyopathy, restrictive cardiomyopathy, left ventricular non-compaction cardiomyopathy or arrhythmogenic right ventricular cardiomyopathy), ventricular arrhythmia and/or cardiac conduction disease including atrioventricular bock (1, 2). Inheritance is typically autosomal dominant but cases of autosomal recessive inheritance have also been described (3, 4). Many pathogenic *DES* variants, through a toxic gain of function, result in intracellular accumulation of desmin aggregates, which are thought to contribute to disease pathophysiology (5, 6). However, not all pathogenic *DES* variants produce aggregates and it has been hypothesized that both loss of function and toxic gain of function may be contributory disease mechanisms (7).

Here we present three desminopathy cases that illustrate the phenotypic heterogeneity of the disease.

## 2. Case series

### 2.1. Case 1

**Figure 1:**
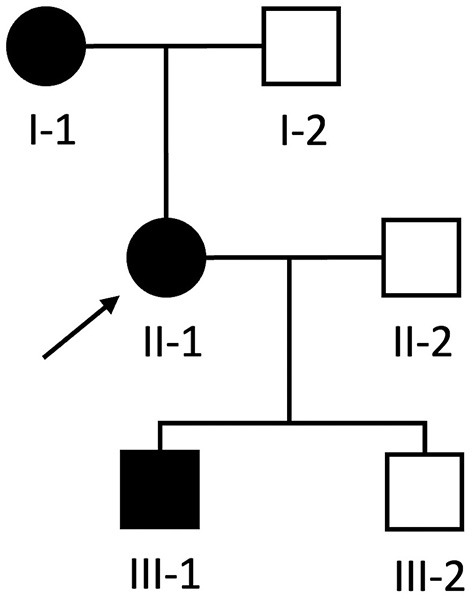
Pedigree of Case 1 (II-1) and Case 2 (III-1). Proband is indicated by the black arrow. Black and white shapes indicate clinically affected and unaffected individuals, respectively. Squares and circles indicate males and females, respectively.

A 54-year-old woman (individual II-1, Figure 1) was referred to our neurology clinic with progressive proximal upper and lower limb weakness over the preceding year. She had a pacemaker inserted at age 30 for complete heart block, previous atrial flutter requiring ablation at age 50 and ongoing paroxysmal atrial fibrillation for which she took amiodarone. Her mother (individual I-1, Figure 1) also required a permanent pacemaker at age 35 for complete heart block.

On examination, there was mild (Medical Research Council grade 4/5), symmetric weakness of shoulder abduction and hip flexion bilaterally with intact reflexes and a normal sensory examination.

Creatine kinase (CK) was mildly elevated at 199 U/L (normal range 34–145 U/L). Electromyography (EMG) revealed early recruitment of myopathic units in proximal muscles. Electrocardiogram (ECG) revealed regular *P* waves and ventricular pacing. Transthoracic echocardiogram (TTE) showed normal left ventricular size and systolic function (ejection fraction 51% by Simpson's biplane method).

**Figure 2:**
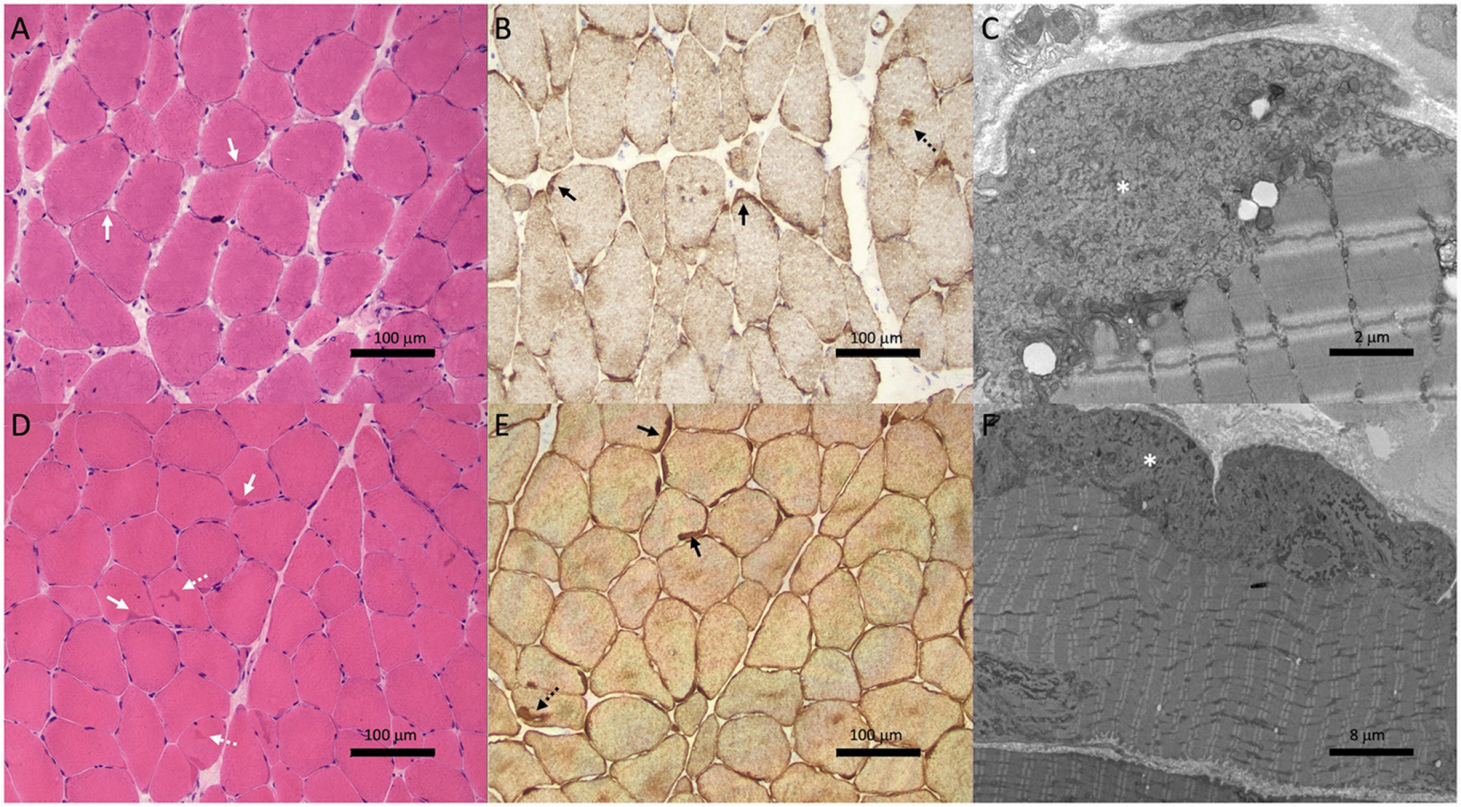
Muscle biopsies from Case 1 (II-1; **A**–**C**) and Case 2 (III-1; **D**–**F**) showed features of myofibrillar myopathy. Hematoxylin & eosin staining **(A, D)** showed subtle subsarcolemmal (solid white arrows) and central basophilic hyaline inclusions (dotted white arrows). Variation in muscle fiber size is also noted in patient II-1 **(A)**. Desmin immunohistochemistry **(B, E)** revealed subsarcolemmal (solid black arrows) and central (dotted black arrows) aggregates. Electron microscopy **(C, F)** showed subsarcolemmal osmiophilic granulofilamentous material (asterisk).

Biopsy of the right deltoid revealed marked variation in muscle fiber diameter (ranging from atrophic to hypertrophic), patchy increase in internal nuclei and scattered fibers with basophilic granularity and peripheral basophilic lobular change (Figure 2A). Desmin immunohistochemistry revealed subsarcolemmal and focal central desmin-positive aggregates (Figure 2B). Electron microscopy showed widespread, predominantly subsarcolemmal accumulation of osmiophilic granulofilamentous material (Figure 2C). These findings were consistent with a myofibrillar myopathy.

Genetic testing revealed a novel heterozygous deletion in *DES* (c.735+1delG) that alters the invariant dinucleotide GT splice donor site at the start of intron 3. This variant is absent from the gnomAD database [American College of Medical Genetics (ACMG) criteria PM2]. Different pathogenic variants affecting the same nucleotide (c.735+1G>A, c.735+1G>C, and c.735+1G>T) have been previously reported in desminopathy patients and have been shown to result in skipping of exon 3 leading to an in-frame deletion of 32 amino acids from the rod 1B domain (criteria PS1 and PM4) (8–10). Park et al. (11) showed that this truncated version of desmin forms abnormal aggregates within the cytoplasm in a cell culture model and Brodehl et al. (12) found desmin-positive aggregates within cardiomyocytes from explanted myocardial tissue in a patient with an exon 3 skipping variant. Together these studies support the c.735+1delG variant as being likely pathogenic.

During 9 years of follow up, there was mild progression in proximal muscle weakness but no development of structural cardiomyopathy or ventricular arrhythmias.

### 2.2. Case 2

During the workup of patient II-1, her then 18-year-old son (individual III-1, Figure 1) was incidentally found to have a systolic murmur and an abnormal ECG during a routine pre-operative assessment prior to elective nasal septoplasty. He was referred to our cardiology clinic and at that time had no dyspnea, palpitations, chest pain or syncope and there was no weakness on neurological examination.

**Figure 3:**
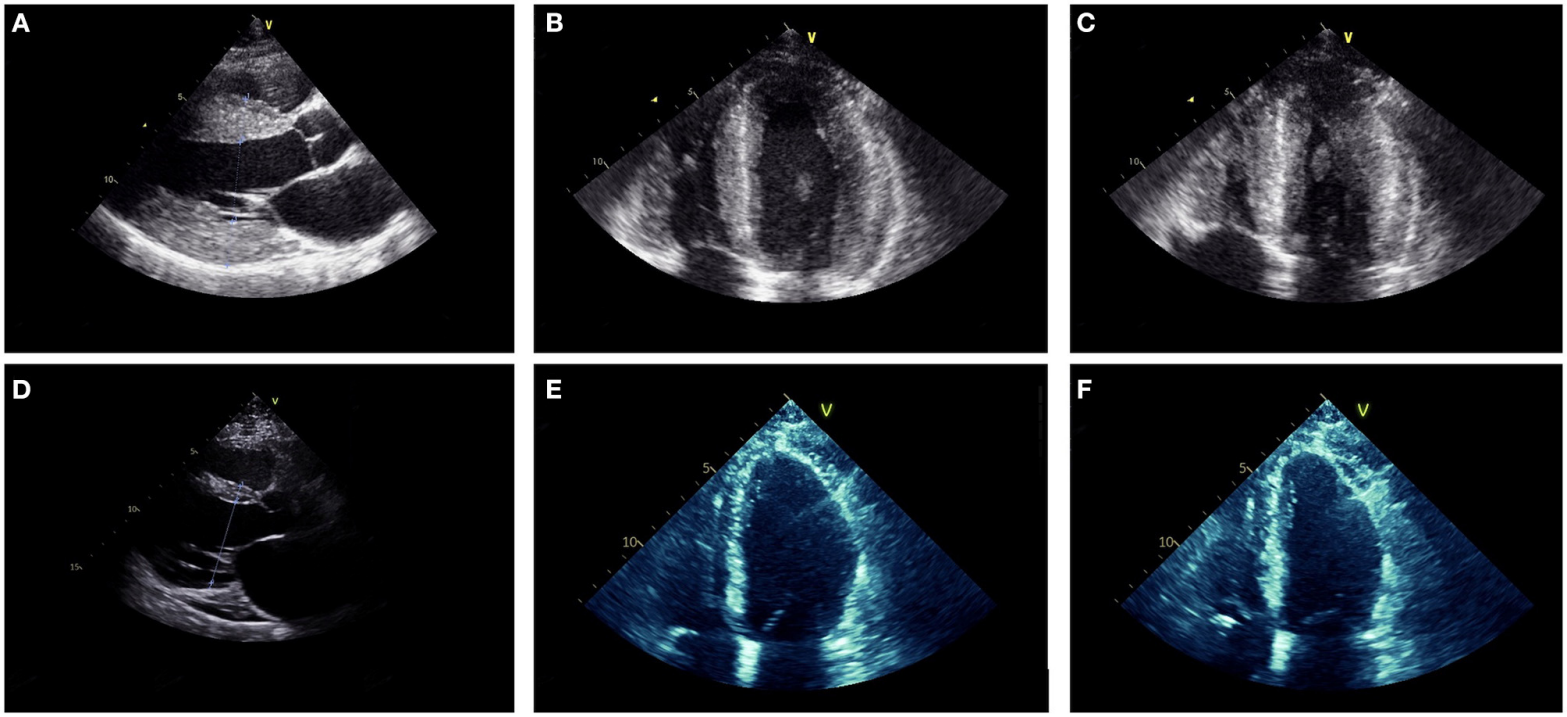
Parasternal long axis **(A, D)**, mid-diastolic apical four chamber **(B, E)** and end-systolic apical four chamber **(C, F)** transthoracic echocardiogram images from Case 2 at ages 18 **(A–C)** and 27 **(D–F)**. At age 18, the ventricular (LV) wall was concentrically thickened (interventricular septum 20 mm, posterior LV wall 20 mm) with vigorous systolic function (LV ejection fraction was 60–65% by Simpson's biplane method). At age 27, the LV wall was thin (interventricular septum 10 mm, LV posterior wall 3 mm) and there was moderate-severe LV dysfunction (LV ejection fraction 35–40% by Simpson's biplane method).

**Figure 4:**
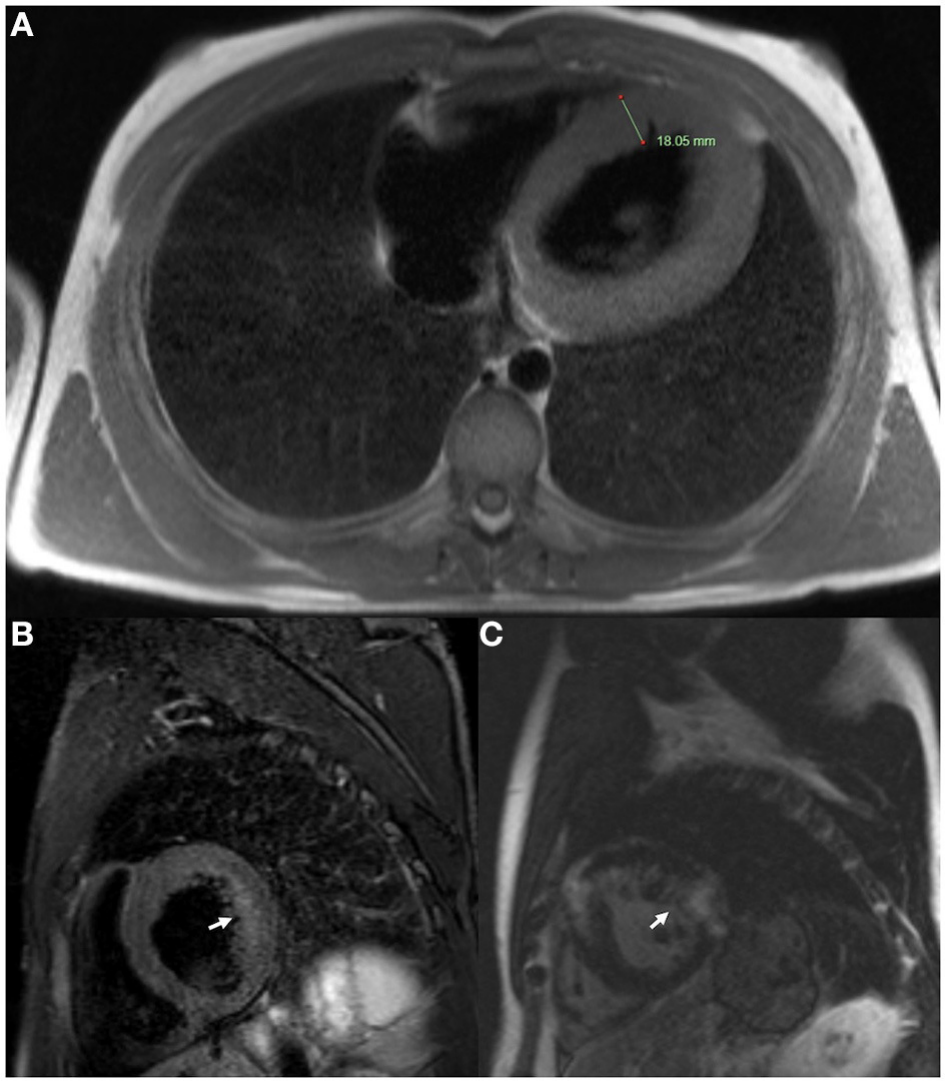
Cardiac magnetic resonance imaging of Case 2 (III-1) at age 18. **(A)** Axial T2 sequence showed increased left ventricular wall thickness (18.05 mm). **(B)** Short axis T2 fat saturation sequence showed increased signal in the mid ventricular wall (arrow). **(C)** Short axis T1 post-gadolinium sequence showed delayed gadolinium enhancement within the ventricular myocardium (arrow).

ECG revealed large QRS amplitudes and T-wave inversions in anterolateral leads suggestive of left ventricular hypertrophy but there was no evidence of conduction system disease. TTE revealed concentrically increased left ventricular wall thickness (up to 20 mm) with preserved systolic function (ejection fraction 60–65% by Simpson's biplane method) and mild-moderate diastolic dysfunction (Figures 3A–C). Holter monitor revealed sinus rhythm with nine sinus pauses >2 s (the longest being 3.4 s), which were asymptomatic. Cardiac magnetic resonance imaging (MRI) demonstrated increased left ventricular wall thickness predominantly affecting the anteroseptal and anterolateral walls (Figure 4A). There was increased mid-wall T2 signal and delayed post-gadolinium enhancement within the thickened segments indicating myocardial oedema and fibrosis (Figures 4B, C). CK was normal at 139 U/L.

Given that the clinical phenotype differed from the predominant conduction disease seen in his mother and grandmother, a biopsy of the right quadriceps was performed. This showed findings consistent with a myofibrillar myopathy with frequent subsarcolemmal hyaline inclusions and occasional centrally-placed inclusions (Figure 2D) which stained positively on desmin immunohistochemistry (Figure 2E). Electron microscopy revealed the same osmiophilic granulofilamentous ultrastructure seen in his mother's biopsy (Figure 2F). Genetic testing showed he carried the familial c.735+1delG *DES* variant.

At age 21, he developed intermittent presyncope and a Holter monitor revealed several runs of non-sustained ventricular tachycardia (longest run 17 beats) prompting insertion of an implantable cardioverter-defibrillator (ICD). Concurrently, sequential TTEs documented progressive reduction in left ventricular wall thickness (Figures 3D–F) and decline in left ventricular systolic function such that ejection fraction was 35–40% with global hypokinesis by age 27. At age 27 he had multiple episodes of ventricular tachycardia, which were terminated by defibrillation from his ICD. Serial ECGs also showed progressive conduction system disease with prolongation of the PR interval to 280 ms and QRS duration to 170 ms at age 27. Despite the biopsy finding of myofibrillar myopathy at age 18, there has been no development of clinically appreciable skeletal muscle weakness during follow up.

### 2.3. Case 3

A 48-year-old male presented with a 5-year history of facial and proximal upper and lower limb weakness, mild scapular winging and bilateral, asymmetric foot drop. CK was elevated at 712 U/L. His mother had a similar phenotype and both were initially clinically diagnosed with facioscapulohumeral dystrophy, however testing of chromosome 4 DZ4Z repeat length was normal. There was no family history of cardiac disease.

While awaiting further genetic testing to return, he presented acutely to hospital with sudden onset of dizziness and dyspnea. He was found to be in ventricular tachycardia and was successfully cardioverted to sinus rhythm. TTE showed a dilated cardiomyopathy with left ventricular ejection fraction of 35–40% (by Simpson's biplane method) and severe inferolateral hypokinesia. An ICD was implanted.

Genetic testing revealed a heterozygous missense c.5G>T (p.Ser2Ile) variant in the desmin head domain that has been previously reported in multiple patients with myofibrillar myopathy (2, 10, 13). Missense variants are a common cause of disease in the desminopathies (ACMG criteria PP2) (14). The Ser2 residue is conserved in mammals and substitution of serine residues in the head domain for hydrophobic residues (such as isoleucine) appears to be a common pattern amongst pathogenic head domain variants (criteria PM1) (15). This c.5G>T variant is absent from gnomAD (criteria PM2) and is predicted to be pathogenic by several *in silico* meta-scores including BayesDel and MetaRNN (criteria PP3). The p.Ser2Ile protein has been shown to form irregular, entangled intermediate filaments in a cell culture model (PS3) (15). As such, this variant is considered to be likely pathogenic.

During 5 years of follow up, the patient has had recurrent episodes of ventricular tachycardia terminated by the ICD.

## 3. Discussion

In this report we present three cases that highlight the well-described clinical heterogeneity of the desminopathies. Despite Case 1 and 2 harboring the same familial variant, Case 1 presented with severe cardiac conduction system disease in early adulthood followed several decades later by a mild limb-girdle pattern of weakness in middle age, whereas Case 2 presented with asymptomatic hypertrophic cardiomyopathy identified in his teenage years which progressed over a few years to dilated cardiomyopathy with ventricular arrhythmias requiring ICD implantation and development of mild conduction system disease. In contrast to Cases 1 and 2 where cardiac symptoms were the initial manifestation of disease, Case 3 initially presented with a combination of facial, scapular and both proximal and distal limb weakness in middle age and then several years later manifested with symptomatic dilated cardiomyopathy and ventricular arrhythmia requiring ICD implantation.

Genotype-phenotype correlations explain some of the phenotypic variability seen in the desminopathies. A systematic review performed by van Spaendonck-Zwarts et al. (14) reviewed 159 patients with 40 different *DES* variants and found that isolated neurologic phenotypes occurred most commonly with variants in the rod 2B domain whereas isolated cardiac phenotypes occurred most commonly with variants in the head or tail domains (14, 16). However, for patients with both neurological and cardiological features, the location of the variant did not seem to predict whether cardiac or neurological features manifested first. More recently, variants in the rod 1B domain (such as the variant in Case 1 and 2) have been linked to a higher likelihood of early onset cardiomyopathy compared to variants in other domains (17). Some of the genotype-phenotype correlation may be explained by the propensity of different *DES* variants to cause loss of function and/or toxic gain of aggregation function (5–7, 17). For example, variants in the desmin tail domain have less propensity to form aggregates (7, 18). The molecular effect of specific variants is also modified by whether they are inherited in isolation (i.e., dominantly) or along with another abnormal allele (i.e., recessively), explaining why phenotype may correlate with mode of inheritance. For example, many dominant gain of function variants present in adulthood whereas some recessive loss of function variants present with severe disease in infancy (3).

Previous attempts at genotype-phenotype correlations may be confounded by several issues relating to the retrospective and cross-sectional nature of these analyses. Firstly, both skeletal myopathy and cardiomyopathy may be present but asymptomatic in an individual, as demonstrated by Case 2 who had biopsy-proven skeletal muscle involvement but no clinical muscle weakness and Case 3 who had dilated cardiomyopathy that had presumably been present for some time before finally manifesting as ventricular tachyarrhythmia. Secondly, the cardiac phenotype may change over time as demonstrated by Case 2 where the initially asymptomatic hypertrophic cardiomyopathy progressed to symptomatic dilated cardiomyopathy with ventricular tachycardia over several years. Interestingly Gudkova et al. (8) report an almost identical presentation of an initially asymptomatic teenage male with a c.735+1G>A variant who, like Case 2, was identified to have a hypertrophic cardiomyopathy on an incidental basis and then over time progressed to a restrictive and then, finally, a dilated cardiomyopathy with development of frequent ventricular tachycardia. Furthermore, although the study by van Spaendonck-Zwarts et al. (14) did not analyse change in cardiac phenotype over time in individual patients, they did find that, at a group level across all different *DES* variants, both restrictive and hypertrophic cardiomyopathies were diagnosed at significantly earlier ages than dilated cardiomyopathy (mean age 28 vs. 33 vs. 46 years, respectively). The same transformation of cardiac phenotype seen in Case 2 is also seen in a murine *DES* knockout model, which is initially characterized by cardiac hypertrophy with preserved left ventricular systolic function followed at a later stage by ventricular dilatation and impaired ventricular systolic function (5). Prospective registries with systematic and longitudinal assessment of cardiac and neurologic phenotypes at multiple time points would help to address the above-mentioned issues and may yield further insights into genotype-phenotype relationships.

Nevertheless, it remains difficult to explain all components of the observed phenotypic variability based on genotype alone. For example, regarding the distribution and severity of neurological involvement, even accounting for age-related penetrance, patients harboring the same variant may present with different degrees and patterns (e.g., proximal limb/limb-girdle, distal limb, proximal and distal limb, scapuloperoneal, facial, etc.) of muscle weakness (10, 14). Indeed, while Case 3 had facial, scapular and proximal and distal limb weakness by his 40s, a previously reported case with the same p.Ser2Ile variant was reported to have no clinically appreciable skeletal muscles weakness at age 48 (10).

Environmental factors may explain some of the otherwise unexplained phenotypic variability and may also contribute to changes in phenotype over time (19). Desmin expression in skeletal muscle is known to increase following sustained muscle activity in healthy volunteers (20). Furthermore, in a desminopathy skeletal muscle cell culture model generated by Segard et al. (19), thermal, oxidative and mechanical stressors precipitated intracytoplasmic desmin aggregation and this occurred more in cells transfected with desmin with a rod domain variant compared to head and tail domain variants. Separately, in a desminopathy rat model, chronic exercise produced increased desmin aggregation and myofibrillar damage resulting in progressive decline in exercise tolerance over time (21). Collating this data, it is possible then that there is a genotype-environment interaction where environmental stressors, including physical exercise, may upregulate desmin expression and precipitate or accelerate toxic aggregation of desmin and that this may be modulated by the propensity of different *DES* variants toward aggregate formation. However, the effect of different types and levels of intensity of exercise has not been explored thoroughly. Indeed, a low-intensity endurance and resistance regimen has been suggested to be safe and even beneficial in a single human desminopathy case report (22).

Gender is another potential contributor to phenotypic variability and may explain some of the differences seen between Cases 1 and 2. The meta-analysis by van Spaendonck-Zwarts et al. (14) found that, across all the pathogenic *DES* variants analyzed, cardiomyopathy was more common in males compared to females (54 vs. 36%) (14). Furthermore, within large desminopathy families with the same variant, cardiomyopathy has been shown to be more frequent, earlier in onset and more severe, often presenting with sudden cardiac death, in males compared to females (23, 24). Our report of early-onset structural cardiomyopathy with recurrent ventricular arrhythmia as the major presenting feature in a male (Case 2) compared to later onset skeletal myopathy (albeit with early onset cardiac conduction deficits) in his mother in her 50s (Case 1) is consistent with this pattern. Arias et al. (23) hypothesized that this gender effect could be mediated by hormonal factors and/or related to less intense physical activity in female patients (13). Regarding hormonal mechanisms, in a murine model of exercise-induced muscle damage, Komulainen et al. (25) found that female rats exhibited significantly less post-exercise loss of subsarcolemmal desmin expression compared to male rats and hypothesized that this may be due to a protective effect of estrogen on sarcolemmal integrity. Thus, gender-exercise interactions may also modify the desminopathy clinical phenotype.

## 4. Conclusion

Our three desminopathy cases emphasize that this condition can present with various combinations of limb and/or facial muscle weakness, different forms of structural cardiomyopathy, ventricular arrhythmias and/or cardiac conduction deficit and that the exact combination and temporal sequence of these clinical features can vary both between different *DES* variants and also within families with the same variant. It is likely that multiple factors including the location of the specific variant within the *DES* gene, mode of inheritance, environmental stressors and gender interact to produce this phenotypic heterogeneity. We also reconfirmed a previous finding that the cardiac phenotype may evolve over time from an initial hypertrophic cardiomyopathy to a dilated cardiomyopathy at later stages indicating that age at time of clinical assessment is another factor that may contribute to apparent variability in the clinical manifestation of this disorder.

## Data availability statement

The datasets presented in this article are not readily available because of ethical and privacy restrictions. Requests to access the datasets should be directed to PM, pamela.mccombe@uq.edu.au.

## Ethics statement

Ethical review and approval was not required for the study on human participants in accordance with the local legislation and institutional requirements. The patients/participants provided their written informed consent to participate in this study.

## Author contributions

DY: drafting of article, study conceptualization, and collection of clinical data. MK, RH, and PM: revision of article, examination of patients, collection of clinical data, and study conceptualization. SP, RD, and SB: revision of article, examination of patients, and collection of clinical data. MD: revision of article and genetic analysis. TR: revision of article, histology, and electron microscopy. All authors contributed to the article and approved the submitted version.
